# 

**Published:** 1854-02

**Authors:** 


### RECEIPTS

### FOR THE JOURNAL DURING THE MONTH OF FEBRUARY.

**Illinois.**

| | |
|---|---|
| Dr. Parker, | $4 00 |
| " Scammon, | 2 00 |
| " Sim, | 2 00 |
| " Palmer, | 2 00 |
| " Lynn, | 3 00 |
| " Quinlan | 2 00 |
| " Blount, | 2 00 |
| " B. G. Stephens, | 6 00 |
| " D. Hess, | 2 00 |
| " Wm. C. Snyder, | 2 00 |
| " S. H Vredenburgh, | 4 00 |
| " L. W. Warren, | 4 00 |

**Indiana.**

| | |
|---|---|
| Dr. J. Gregory, | $2 00 |
| " David Hutchinson, | 2 00 |
| " H. P. Howes, | 2 00 |
| " Charles Badger, | 4 00 |
| " R. Q. Wilson, | 2 00 |

**Wisconsin.**

| | |
|---|---|
| Dr. Wm. Cargen, | $2 00 |
| " M'Kinlay, | 2 00 |
| " W. R. Godfrey, | 3 00 |

**Ohio.**

| | |
|---|---|
| Dr. R. P. Lamb, | $2 00 |

